# Telephone survey of private patients' views on continuity of care and registration with general practice in Ireland

**DOI:** 10.1186/1471-2296-8-17

**Published:** 2007-03-30

**Authors:** Patricia Carmody, David L Whitford

**Affiliations:** 1 Fairview Family Practice, 17 Fairview Strand, Dublin 3, Ireland; 2 Department of Family Medicine, Royal College of Surgeons in Ireland – Medical University of Bahrain, PO Box 15503, Kingdom of Bahrain

## Abstract

**Background:**

The desire of patients for personal continuity of care with a General Practitioner (GP) has been well documented, but not within non-registered private patients in Ireland. This study set out to examine the attitudes and reported behaviours of private fee-paying patients towards continuity of GP care and universal registration for patients.

**Methods:**

Cross-sectional telephone survey of 400 randomly chosen fee-paying patients living within County Dublin. There is no formal system of registration with a GP for these patients. Main outcomes were attendance of respondents at primary health care facilities and their attitudes towards continuity of care and registration with a GP. Data was analysed using descriptive statistics and using parametric and non-parametric tests of association. Pearson correlation was used to quantify the association between the described variables and attitudes towards continuity and registration with a GP. Variables showing significance at the 5% level were entered into multiple linear regression models.

**Results:**

97% of respondents had seen a GP in the previous 5 years. The mean number of visits to the GP for respondents was 2.3 per annum. 89% of respondents had a regular GP and the mean length of time with their GP was 15.6 years. 96% preferred their personal medical care to be provided within one general practice. 16% of respondents had consulted a GP outside of their own practice in the previous year. They were more likely to be female, commute a longer distance to work or have poorer health status. 81% considered it important to be officially registered with a GP practice of their choice.

**Conclusion:**

Both personal and longitudinal continuity of care with a GP are important to private patients. Respondents who chose to visit GPs other than their regular GP were not easily characterised in this study and individual circumstances may lead to this behaviour. There is strong support for a system of universal patient registration within general practice.

## Background

Continuity of care is traditionally considered a core value of general practice [1]. There are different aspects of continuity of care, with three of these aspects having been organised into a hierarchy ranging from the availability of accurate information from one health care encounter to another (informational continuity), through a pattern of health care utilization at a particular site of care (longitudinal continuity), to a personal doctor-patient relationship characterized by loyalty and trust (interpersonal continuity)[2,3]. Interpersonal continuity holds particular importance for many general practitioners (GPs) and has been described as an ongoing therapeutic relationship between patient and GP, with the patient looking to the practitioner as their most valued source of care[4]. Patients consider it to be important[5] and it is associated with increased patient satisfaction[6] and significant improvements in preventive services and hospitalisation[3].

Until recently, over 50% of general practices in Ireland were single handed, a characteristic associated with greater inter-personal continuity of care[7]. Access to general practice for patients is usually rapid. However, the changing face of general practice has been predicted to threaten both longitudinal and personal continuity of care[4,8]. General practice in Ireland is currently undergoing changes under government policy with the aim of establishing a stronger role for the primary care team, a greater team-based approach and an enhanced capacity for primary care in the areas of disease prevention, rehabilitation and personal social services to complement the existing diagnosis and treatment focus[9]. The Government Health Strategy proposes to achieve this through the introduction of an inter-disciplinary team-based approach to primary care provision. These changes may lead to further challenges to the GP-patient relationship, as patients may receive continuity of care from the primary care team as opposed to the individual GP, a concept described elsewhere[10].

Patients in Ireland currently access general practice in two ways. 30% of the population receive means-tested free primary care (known as General Medical Services (GMS)) and are registered with a GP. The remainder of the population are fee-paying private patients and there is no requirement for registration with GPs for this population. Private patients can choose to change GPs regularly or can have any number of GPs at one time. There are anecdotal reports of private patients moving regularly between different GPs. There are also suggestions that the lack of a registered list of private patients has the effect of reducing the amount of anticipatory care carried out by GPs due to the difficulty of defining the practice population. Utilisation of general practice by private patients has been studied in Ireland, but the information gathered has looked primarily at visiting rates[11] and there have been no studies looking at attitudes towards continuity of care or desire for personal registration with a GP.

This study set out to examine the attitudes and reported behaviours of private fee-paying patients towards continuity of GP care and universal registration for patients. In addition, we sought to identify parameters that might define a practice population in the absence of universal patient registration.

## Methods

Three focus groups[12] were employed in order to identify themes surrounding the utilisation of general practice. A sample of private patients who had consulted in two Dublin practices in the previous year was invited to attend. An independent researcher led the interviews, which were electronically recorded and lasted approximately one hour.

Several themes developed during the analysis of the interviews by the independent researcher. Data were assembled around these themes (See Additional file 1) which were used in the construction of the survey instrument by the authors. The developed questionnaire was then adapted by MORI for use in a telephone survey (See Additional file 2).

A random sample was drawn from the population of County Dublin (1.2 million population). A sample size of 384 patients was estimated to give a 95% chance of being within 5% of the true result based on this population size. The sample was drawn through random generation of telephone numbers.

Ipsos MORI was commissioned to carry out 400 computer assisted telephone interviews using the survey instrument. Respondents were excluded if they were aged less than 18 years, aged over 69 years (patients aged 70 years or over are covered by the GMS scheme), received free medical care through the GMS scheme or had never attended a GP in Ireland. Soft quotas on age and gender were given in order to achieve a spread over these parameters. The interviews took ten minutes and were conducted over a two-week period.

Background information was collected on the respondents' age, gender, ethnicity, marital status, educational status, social class, work status, and health status. Further questions examined the patients' attendance at health care facilities and their attitudes towards continuity of care and registration with a GP.

Data was analysed using descriptive statistics. Data analysis was with SPSS 14.0 using parametric and non-parametric tests of association including Students t and chi-squared tests. P < 0.05 was taken as significant. Pearson correlation was used to quantify the association between the described variables and attitudes towards continuity and registration with a GP. A Pearson correlation coefficient and significance test for the correlation coefficient was calculated using SPSS 14.0. Variables showing significance at the 5% level were entered into multiple linear regression models with forward selection.

## Results

**Figure 1:**
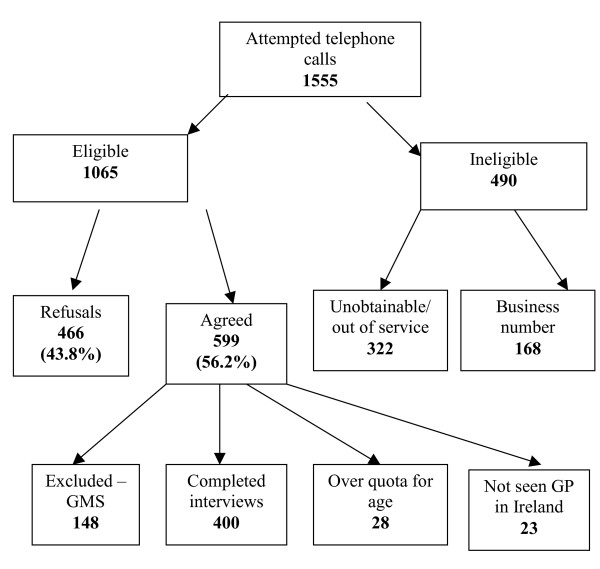
Conduct of telephone survey.

400 people completed the telephone survey. 56.2% of residential contacts agreed to take part (Figure 1).

### Utilisation of general practice

95% (400/423) of people contacted had attended a GP in Ireland. The main reason for not having seen a GP in Ireland was recent arrival in the country (14/23 (61%)). 309/400 (77%) respondents had attended a GP in the previous year, and 364/400 (91%) in the previous 2 years. Only 10 (2.5%) respondents had not seen a GP in the previous five years. Respondents with a disability or illness were more likely to have seen a GP in the previous year (90%v74%, X^2 ^= 10.06, 1df, p = 0.001). The mean number of visits per annum to the GP for all respondents was 2.3 visits. Female respondents attended more frequently then their male counterparts (2.6 v 2.07, F = 5.9, 1df, p = 0.016).

### Continuity of care

357 (89%) respondents considered themselves to have a regular GP. Table 1 shows older age to be associated with having a regular GP. Regression analysis showed older age and more recent attendance at a GP to be predictive of having a regular GP, accounting for 12% of the variance (p < 0.001). The mean length of time spent with the same GP was 15.6 years. 310 (78%) respondents stated they receive their personal medical care from a regular GP; while a further 72 (18%) respondents receive their care from GPs within the same practice. 85% of respondents considered it fairly or very important to see the same GP at each visit.

**Table 1:** Association of respondent characteristics with having a regular GP

	Do you have a GP you consider to be your regular GP?				
Characteristic		Number	Yes	No	Statistic

Age (n = 400)	18–34 years	105	81.9%	18.1%	X^2 ^= 13.23, 2df, p = 0.001*
	35–54 years	186	88.7%	11.3%	
	55–69 years	109	97.2%	2.8%	
Gender (n = 400)	Male	133	89.5%	10.5%	X^2 ^= 0.01, 1df, p = 0.92
	Female	267	89.1%	10.1%	
Marital status (n = 400)	Single	138	86.2%	13.8%	X^2 ^= 2.0, 1df, p = 0.16
	Married/cohabiting	262	90.8%	9.2%	
Children in household (n = 400)	Yes	132	90.2%	9.8%	X^2 ^= 0.17, 1df, p = 0.68
	No	268	88.8%	11.2%	
Social class (n = 399)	AB	140	90%	10%	X^2 ^= 0.29, 2df, p = 0.87
	C	195	89.2%	10.8%	
	DE	64	87.5%	12.5%	
Employment status (n = 397)	Working	285	89.1%	10.9%	X^2 ^= 0.002, 1df, p = 0.96
	Unemployed	112	89.3%	10.7%	
Commute distance (n = 394)	<2 km	143	89.5%	10.5%	X^2 ^= 0.007, 1df, p = 0.93
	>2 km	251	89.2%	10.8%	
Health status (n = 400)	Good	356	89%	11%	X^2 ^= 0.14, 1df, p = 0.71
	Poor	44	90.9%	9.1%	
Long term illness or disability (n = 398)	Yes	82	95.1%	4.9%	X^2 ^= 3.76, 1df, p = 0.052
	No	316	87.7%	12.3%	

*Significant at 5% level

The reasons for choosing their present GP fell into two main categories: convenience to home or work and personal recommendation from family or friend. Respondents indicated that they would consider a GP to be their regular GP after a mean of four visits to the same GP.

Interviewees were asked whom they would prefer to receive their personal medical care from. 326 (82%) respondents expressed a preference for their personal medical care to be provided by a regular GP, whilst 54 (14%) opted for a GP within the same practice and only 20 (5%) for care outside of general practice. Regression analysis showed younger age to be most predictive of a preference for care outside of a regular GP practice (p < 0.001). Factors important to respondents in seeing a regular GP are shown in Table 2.

**Table 2:** Factors important to respondents in continuing to see a regular GP (n = 400)

Factor	Number (%) of prime importance*	Subfactor	Number (%) of prime importance	Number (%) of subsequent mentions
Relational factors	157 (39%)	Good communication	60 (15%)	100 (25%)
		Trust in GP	41 (10%)	89 (22%)
		Long term relationship	22 (6%)	48 (12%)
		Gives me time	21 (6%)	40 (10%)
		Friendly manner of GP	9 (2%)	20 (5%)
		Gender of GP	4 (1%)	12 (3%)
Informational factors	77 (19%)	Knows my past medical history	77 (19%)	103 (26%)
Access factors	75 (19%)	Able to make appointment	38 (10%)	55 (14%)
		Location of surgery	21 (6%)	39 (10%)
		Convenient hours for work	16 (4%)	30 (7.5%)
Quality/service factors	50 (12.5%)	Quality service	17 (4%)	33 (8%)
		Keeps up to date	11 (3%)	15 (4%)
		Thorough examination/diagnosis	8 (2%)	16 (4%)
		Modern facilities	7 (2%)	13 (3%)
		Cost	7 (2%)	19 (5%)
Others	10 (2.5%)			20 (5%)
Don't know	31 (8%)			31 (8%)

*Prime importance refers to the first factor mentioned on being asked the question.

59/378 (16%) respondents had attended a GP outside of their own practice in the previous year. Table 3 indicates that respondents who were younger, female and with poorer health status were more likely to be associated with attending a GP other than their regular GP practice. Regression analysis showed female gender, poorer health status and longer commuting distances to work to be most predictive of attending another GP (p = 0.01). Patients expressing a preference for a GP within the same practice were as likely to see a GP outside of their own practice as patients who expressed a preference to attend any practice (15% v 20%, X^2 ^= 1.0, 1df, p = 0.3). The main reason given for attending a GP outside of their practice was that their regular GP was not available (n = 21 (36%)). Other reasons included recommendation from others (25%), convenience to work or home (22%), cost (10%), sensitive medical problem (3%) and problems with their own GP (3%). Only 9 (2%) respondents had attended a GP cooperative and 22 (6%) a GP deputising service in the previous year. 66 (17%) respondents had used an Accident & Emergency Department with the majority of these (71%) only attending once in the previous year. Frequent attendance in general practice was associated with attending these other services (p= 0.009).

**Table 3:** Association of respondent characteristics with attendance at other GP practices

	In addition to your regular GP practice, have you attended any other GPs or practices in the last 12 months?				
Characteristic		Number	Yes	No	Statistic

Age (n = 378)	18–34 years	97	22.7%	77.3%	X^2 ^= 6.75, 2df, p = 0.034*
	35–54 years	175	15.4%	84.6%	
	5569 years	106	9.4%	90.6%	
Gender (n = 378)	Male	124	9.7%	90.3%	X^2 ^= 4.93, 1df, p = 0.026*
	Female	254	18.5%	81.5%	
Marital status (n = 378)	Single	130	18.5%	81.5%	X^2 ^= 1.22, 1df, p = 0.27
	Married/cohabiting	248	14.1%	85.9%	
Children in household (n = 378)	Yes	126	14.3%	85.7%	X^2 ^= 0.25, 1df, p = 0.61
	No	252	16.3%	83.7%	
Social class (n = 377)	AB	135	16.3%	83.7%	X^2 ^= 1.48, 2df, p = 0.48
	C	184	16.8%	83.2%	
	DE	58	10.3%	89.7%	
Employment status (n = 375)	Working	271	17%	83%	X^2 ^= 1.13, 1df, p = 0.18
	Unemployed	104	12.5%	87.5%	
Commute distance (n = 372)	<2 km	136	11%	89%	X^2 ^= 3.04, 1df, p = 0.08
	>2 km	236	17.8%	82.2%	
Health status (n = 378)	Good	339	13.9%	86.1%	X^2 ^= 7.6, 1df, p = 0.0006*
	Poor	39	30.8%	69.2%	
Long term illness or disability (n = 376)	Yes	77	20.8%	79.2%	X^2 ^= 1.9, 1df, p = 0.17
	No	299	14.4%	85.6%	

*Significant at 5% level

**Figure 2:**
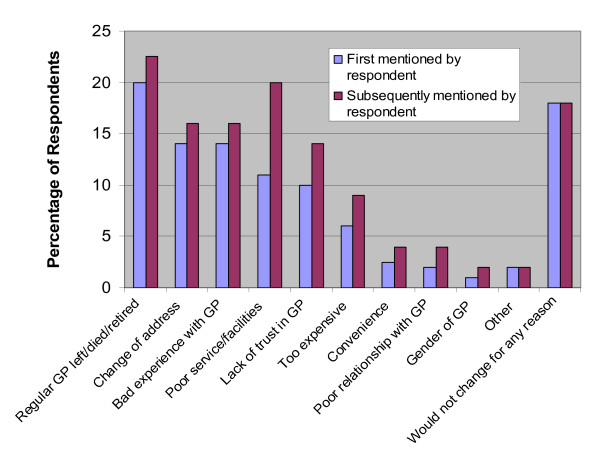
Factors that would encourage respondents to change their General Practitioner (n = 400).

When asked about the likelihood of changing GPs in the next 12 months 89% indicated this was very or fairly unlikely. All respondents were asked what drivers would encourage them to change GP (Figure 2). The commonest reasons were the GP leaving the practice due to retirement or death and a change of address.

### Registration

The majority of respondents (324 (81%)) considered it is important to be officially registered with a GP practice of their choice, with 58% indicating it is 'very important' and 23% 'fairly important'. After prompting with possible benefits of registration (including continuity of care, better knowledge of medical history and invitations to screening programmes) 369 (92%) considered it fairly or very important to be registered with a GP practice of their choice. Table 4 indicates that female patients were more likely to support registration with a GP practice. Regression analysis showed lower educational level to be most predictive of thinking registration would be beneficial (p = 0.002).

**Table 4:** Association of respondent characteristics with attitude towards registering with a GP practice

	Would you think it is important or not to be officially registered with one GP practice of your choice?					
Characteristic		Number	Very/fairly	Neither	Not very/fairly	Statistic

Age (n = 400)	18–34 years	105	77.1%	4.8%	18.1%	X^2 ^= 3.73, 4df, p = 0.44
	35–54 years	186	80.1%	4.8%	15.1%	
	55–69 years	109	86.2%	4.6%	9.2%	
Gender (n = 400)	Male	133	73.7%	6.8%	19.5%	X^2 ^= 6.95, 2df, p = 0.03*
	Female	267	84.6%	3.7%	11.6%	
Marital status (n = 400)	Single	138	78.3%	4.3%	17.4%	X^2 ^= 1.7, 2df, p = 0.42
	Married/cohabiting	262	82.4%	5%	12.6%	
Children in household (n = 400)	Yes	132	81.8%	4.5%	13.6%	X^2 ^= 0.09 2df, p = 0.96
	No	268	80.6%	4.9%	14.6%	
Social class (n = 399)	AB	140	77.9%	7.1%	15%	X^2 ^= 3.43, 4df, p = 0.49
	C	195	83.6%	3.1%	4.7%	
	DE	64	79.7%	13.3%	15.6%	
Employment status (n = 397)	Working	285	80.7%	4.9%	14.4%	X^2 ^= 0.11, 2df, p = 0.95
	Unemployed	112	82.1%	4.5%	13.4%	
Commute distance (n = 394)	<2 km	143	85.3%	4.2%	10.5%	X^2 ^= 0.2.58, 2df, p = 0.27
	>2 km	251	78.9%	5.2%	15.9%	
Health status (n = 400)	Good	356	81.7%	4.2%	14%	X^2 ^= 2.28, 2df, p = 0.32
	Poor	44	75%	9.1%	15.9%	
Long term illness or disability (n = 398)	Yes	82	84.1%	4.9%	11%	X^2 ^= 0.95, 2df, p = 0.62
	No	316	80.4%	4.4%	15.2%	

*Significant at 5% level

## Discussion

This study reveals that, even in a health care system in which there is no obligation on fee paying patients to attend the same GP, the vast majority of people choose to have a regular GP, to attend that GP for all their health care, and to remain with the same GP over a long period of time. The population studied considers both personal and longitudinal continuity of care with a GP to be important, and this is indicated by both their expressed attitudes and their reported behaviours. There is clear support for a system of GP registration for private patients in Ireland.

### Strengths and weaknesses of this study

We decided to use a telephone survey for this study as it would be relatively rapid and enable us to easily exclude those respondents who did not fit our categories (patients under the GMS scheme). In addition, the response rates are known to be relatively high in telephone surveys. However, telephone surveys have weaknesses including a selection bias in omitting those who do not have phones and those less likely to use a landline or to be at home. In addition, the survey instrument needs careful design to tailor the questions to a telephone survey. We are aware of the possibility of a response bias in answering the questions on patient registration as the concept was not defined to the patient in the survey instrument. In addition, this was a first pilot study looking at continuity of care in Ireland, but could have explored some of the concepts in greater depth than our survey instrument allowed.

### Comparison with previous studies

The figure of 11% of respondents not having a regular doctor is similar to a previous study in Newfoundland where 15% of the population did not have a regular doctor[13]. A relationship between length of time with a doctor and the value placed on continuity has also been previously established[14]. The mean length of time spent with a GP for this population was 15.6 years and this is reflected in the positive views expressed towards continuity of care. As in other studies younger people were least likely to have adopted attitudes and behaviours supporting continuity of care with a GP[13,15]. This may be related to a different approach towards healthcare utilisation in this group or may be that as a group they are generally healthier, more mobile and more likely to be consulting a GP about acute episodes of illness.

Previous studies have shown frequent users of services outside of a regular GP for primary care to be a psychosocially vulnerable group[16]. In this study, we found that users of other services, including Accident & Emergency and out of hours GP services were also likely to be frequent attendees at their own regular GP.

Attendance rates for the GP were low at a mean of 2.3 visits per year and may reflect the lack of delivery of preventive services to this population. However, this is consistent with previous studies of GP visiting rates among private patients in Ireland, as is the higher visiting rate amongst female patients[17]. However, the proportion of respondents who had visited a GP in the previous year (77%) is higher than in previous studies (65%)[17]. This may be due to this study being carried out on a more urban population. This study supports previous studies in indicating that the overwhelming majority (97%) of the population have seen a GP in the previous five years.

Patients in this study chose their GP on the basis of convenience and personal recommendation. Important factors to the patient in continuing with the same GP are the GP's knowledge of their medical history, good communication with the GP and trust in their GP; factors similar to other studies[18]. Few patients were intending to change GP. The main precipitant for such a change would be the death or retirement of their GP. This would seem an obvious factor but has not been reported in previous studies[19,20]. Other reasons including change of address and breakdown of the relationship with their GP are similar to these previous studies.

### Meaning of this study

It is clear from this study that private patients in Ireland have positive attitudes towards continuity of care with a GP. This has implications for proposed changes in the Irish Healthcare system encouraging registration with a primary care team as opposed to a GP practice.

Only 16% of respondents had visited a GP other than their regular GP in the previous year. We sought to characterise this group but could account for only 4% of the variance, explained by female gender, poorer health and a longer commuting distance to work. However, there was no difference between those who expressed the view that seeing a regular GP was important and those who did not in the likelihood of having consulted a GP outside their own practice in the previous year. The reasons given for attending another GP tended to indicate 'one-off' occurrences as opposed to a regular pattern of behaviour. This suggests that there may not be a distinct group of people who use different GPs on a regular basis but more likely a group of circumstances that encourage this[21].

We sought to define a practice population in the absence of universal registration. It appears that this population of private patients behave as a stable GP population, even in the absence of registration. The majority of patients visit one regular GP or practice over long periods of time. The patients who attend a GP outside of their regular GP are not readily characterised. The question remains as to how GPs can identify a practice population in order to institute population based programmes. We would propose several options:

• As 92% of the fee-paying population have attended a GP in the last two years, it could be argued that any patient having attended a GP in the last two years could be considered as 'registered'. However, it is not clear whether patients who visit a GP once will visit that same GP again.

• Respondents indicated that they would consider a GP their 'regular GP' after a mean of four visits. Patients who had attended four or more times with a GP could be considered as 'registered'. However, when considering invitations for screening, 'low-attendees' would be less likely to be screened.

• Practices could institute their own registration scheme. In exchange for 'registration', the practice could offer continuity of care and invitations to screening. Patients could continue to opt for 'non-registered' appointments.

• General practice could continue to pressure the government for a system of universal registration in order to enhance continuity of care and improve anticipatory care. This study suggests there is little opposition to this idea from patients, particularly when the benefits are explained to the patient. In addition, it appears that a more socially disadvantaged population are most supportive of universal registration. The reasons for this are not clear. However, the more socially disadvantaged group of private patients are particularly vulnerable in being just above the means tested limit for access to general practice. We would argue that this population might be most likely to benefit from universal registration as lack of registration has been linked to lower attendance in primary care by vulnerable populations[22].

### Future research

There is a need in the Irish context to further establish the importance of continuity of care in relationship to other aspects of healthcare delivery such as speed of access. In addition, it is clear that not all patients need or desire the same aspects of care and developing models of health service delivery that are flexible enough to respond to these different needs and desires remains a priority.

## Conclusion

From this study, we can conclude that private patients value a personal relationship with their GP. They choose to have a personal and longitudinal continuity of care with their GP. There is little resistance to universal patient registration with GPs in this population and this should be a government priority for the health service. This would enable GPs to define a practice population to which they can deliver appropriate anticipatory care.

## Competing interests

The author(s) declare that they have no competing interests.

## Authors' contributions

PC conceived of the study, and participated in its design and coordination and helped to draft the manuscript. DW participated in the design of the study, performed the statistical analysis and drafting of the manuscript. All authors read and approved the final manuscript.

## Pre-publication history

The pre-publication history for this paper can be accessed here:



## Supplementary Material

Additional file 1 Qualitative analysis. Themes emerging from analysis of focus groups Click here for file

Additional file 2 Telephone Questionnaire. Questionnaire developed and used for telephone survey Click here for file
