# Supplementary material for: Telephone survey of private patients' views on continuity of care and registration with general practice in Ireland
Source: BMC Fam Pract. 2007 Mar 30;8:17. doi: 10.1186/1471-2296-8-17 (PMC1851962; doi:10.1186/1471-2296-8-17)
Supplement: Additional file 1 — Qualitative analysis. Themes emerging from analysis of focus groups [file 1471-2296-8-17-S1.doc]

**Appendix 1 – Qualitative analysis**

Several themes emerged from the focus groups when discussing continuity of care. Major themes were:

- Trust in GP
- Relationship with GP
- GP attitude
- GP responsibility
- Influences on choice of GP
- Convenience of access
- Choice
- Waiting times
- Attending A&E
- Preventive services

The questionnaire development was adapted from the patient views expressed around several of these themes.
