# Supplementary material for: Telephone survey of private patients' views on continuity of care and registration with general practice in Ireland
Source: BMC Fam Pract. 2007 Mar 30;8:17. doi: 10.1186/1471-2296-8-17 (PMC1851962; doi:10.1186/1471-2296-8-17)
Supplement: Additional file 2 — Telephone Questionnaire. Questionnaire developed and used for telephone survey [file 1471-2296-8-17-S2.doc]

**Appendix 2 Telephone Questionnaire**

# Royal College of Surgeons in Ireland (RCSI)

# General Public Survey

# Ipsos MORI

**Internal Specifications:**

n=400

Soft quotas on age and gender

Age bands soft quotas:

18-34 yrs – n= 130 (minimum of 100)

35-54 yrs – n= 130 (minimum of 100)

55-69 yrs – n= 130 (minimum of 100)

Gender soft quotas:

Male – n=120 (30%)

Female – n=280 (70%)

Location (collected via postcode): Randomly sampled from Dublin city and county area – Field to ensure a good spread of postcodes. Monitor Dublin City / Dublin County split.

**Introduction:**

Good morning/afternoon, my name is ……………………….. from Ipsos MORI, an independent market research company. We are undertaking a survey on behalf of the Department of General Practice at the Royal College of Surgeons in Ireland. Can I ask you a few questions?

**IF ASKED ABOUT TIME**: This will take about 8-9 minutes, is it convenient to talk now or can I call you back at a more convenient time?

I just need to ask a few questions first.

| S1 | Have you attended a GP in Ireland? **READ OUT** | Yes | 1 | Continue |
| --- | --- | --- | --- | --- |
|  |  | No | 2 | Go to Q18-19 then Close & Recruit Substitute |

| S2 | Do you have a GMS (General Medical Services) card or a ‘doctor only’ card? **DO NOT** **READ OUT** | Yes – have one or both cards | 1 | Close & Recruit Substitute |
| --- | --- | --- | --- | --- |
|  |  | No - do not have either card | 2 | Continue |

| S3 | What age are you? **INSERT NO. IN BOX. RECRUIT AGES 18-69 YRS ONLY.** |  |  | **TERMINATE IF UNDER 18 OR 70+ OR REFUSED** |
| --- | --- | --- | --- | --- |

| S4 | Record gender **DO NOT READ OUT** | Male | 1 | Continue and soft check quota |
| --- | --- | --- | --- | --- |
|  |  | Female | 2 | Continue and check soft quota |

| S5 | Could you tell me in which postal district of Dublin you live? **DO NOT** **READ OUT** | D1 | 1 | MONITOR SPREAD OF POSTCODES – CITY VS. COUNTY |
| --- | --- | --- | --- | --- |
|  |  | D2 | 2 |  |
|  |  | D3 | 3 |  |
|  |  | D4 | 4 |  |
|  |  | D5 | 5 |  |
|  |  | D6 | 6 |  |
|  |  | D7 | 7 |  |
|  |  | D8 | 8 |  |
|  |  | D9 | 9 |  |
|  |  | D10 | 10 |  |
|  |  | D11 | 11 |  |
|  |  | D12 | 12 |  |
|  |  | D13 | 13 |  |
|  |  | D14 | 14 |  |
|  |  | D15 | 15 |  |
|  |  | D16 | 16 |  |
|  |  | D17 | 17 |  |
|  |  | D18 | 18 |  |
|  |  | D19 | 19 |  |
|  |  | D20 | 20 |  |
|  |  | D21 | 21 |  |
|  |  | D22 | 22 |  |
|  |  | D23 | 23 |  |
|  |  | D24 | 24 |  |
|  |  | County Dublin | 25 |  |
|  |  | Other | 26 | Terminate |

**Main Survey:**

ASK ALL

| Q1 | When was the last time you attended a GP, was it..?  **READ OUT**  **Single code** | In the last 12 months | 1 | Go to Q2a |
| --- | --- | --- | --- | --- |
|  |  | 1 to 2 years ago | 2 | Go to Q2b |
|  |  | 2 to 3 years ago | 3 | Go to Q2a |
|  |  | 3 to 4 years ago | 4 | Go to Q2b |
|  |  | 4 to 5 years ago | 5 | Go to Q2b |
|  |  | More than 5 years ago | 6 | Go to Q2b |
|  |  | DK / Can’t remember | 0 | Go to Q2b |

**ASK IF VISITED GP IN L12M. IE CODE 1 IN Q1. OTHERS GO TO Q2B**

| Q2a | In the last 12 months, how often have you visited a GP?  **RECORD NUMBER** |  |
| --- | --- | --- |
|  |
|  |
|  |

ASK ALL

| Q2b | Do you have a GP you consider to be your regular GP?  **READ OUT**  **Single code** | Yes | 1 | Go to Q2c |
| --- | --- | --- | --- | --- |
|  |  | No | 2 | Go to Q3 |
|  |  | DK | 0 | Go to Q3 |

**ASK IF HAVE REGULAR GP. IE CODE 1 IN Q2B. OTHERS GO TO Q3**

| Q2c | How long have you been seeing your regular GP? |  |  |  |
| --- | --- | --- | --- | --- |
|  |  | RECORD NO. OF YEARS |  |  |
|  |  | OR NO. OF MONTHS IF < 1 YEAR |  |  |

**ASK ALL**

| Q3 | Thinking about your personal medical care, do you **prefer** this to be provided by..?  **READ OUT 1-5**  **Single code** | A regular GP | 1 |  |
| --- | --- | --- | --- | --- |
|  |  | Any GP within the same practice | 2 |  |
|  |  | Any GP from different practices | 3 |  |
|  |  | Hospital A&E | 4 |  |
|  |  | Hospital Outpatient Services | 5 |  |
|  |  | Other (specify)  …………………………………………………….. | 6 |  |

| Q4a | Which of the following is your personal medical care provided by…?  **READ OUT 1-5**  **Multi code** | A regular GP | 1 |  |
| --- | --- | --- | --- | --- |
|  |  | Any GP within the same practice | 2 |  |
|  |  | Any GP from different practices | 3 |  |
|  |  | Hospital A&E | 4 |  |
|  |  | Hospital Outpatient Services | 5 |  |
|  |  | Other (specify)  …………………………………………………….. | 6 |  |

| Q4b | Which of the following is your personal medical care **mainly** provided by…?  **READ OUT 1-5**  **Single code** | A regular GP | 1 | Go to Q5 |
| --- | --- | --- | --- | --- |
|  |  | Any GP within the same practice | 2 | Go to Q5 |
|  |  | Any GP from different practices | 3 | Go to Q7 |
|  |  | Hospital A&E | 4 | Go to Q7 |
|  |  | Hospital Outpatient Services | 5 | Go to Q7 |
|  |  | Other (specify)  …………………………………………………….. | 6 | Go to Q7 |

ASK IF HAVE REGULAR GP OR PRACTICE. IE CODE 1 OR 2 IN Q4B. OTHERS GO TO Q7

| Q5 | In addition to your regular GP practice, have you attended any other GPs or practices in the last 12 months?  **READ OUT**  **Single code** | Yes | 1 | Go to Q6 |
| --- | --- | --- | --- | --- |
|  |  | No | 2 | Go to Q7 |
|  |  | DK | 0 | Go to Q7 |

**ASK IF HAVE ATTENDED OTHER GPS. IE CODE 1 IN Q5. OTHERS GO TO Q7**

| Q6 | Why did you attend another GP and not your regular GP practice?  **DO NOT PROMPT, MULTI CODE** | Convenience to work | 1 |  |
| --- | --- | --- | --- | --- |
|  |  | Convenience to home | 2 |  |
|  |  | Confidentiality / sensitive medical condition e.g. contraception, STD’s | 3 |  |
|  |  | Recommendation from family or friends | 4 |  |
|  |  | Regular GP not available | 5 |  |
|  |  | Other (specify)  …………………………………………………….. | 6 |  |
|  |  | DK | 0 |  |

**ASK ALL**

| Q7 | Why did you choose your current GP practice?  **DO NOT PROMPT, RECORD FIRST AND OTHER MENTIONS** |  | **First**  **SC** | **Others**  **MC** |  |
| --- | --- | --- | --- | --- | --- |
|  |  | Convenience to home | 1 | 1 |  |
|  |  | Convenience to work | 2 | 2 |  |
|  |  | **Recommendation:** |  | | |
|  |  | Family | 3 | 3 |  |
|  |  | Friends | 4 | 4 |  |
|  |  | Work colleagues | 5 | 5 |  |
|  |  | Other doctors | 6 | 6 |  |
|  |  | Irish medical directory | 7 | 7 |  |
|  |  | Internet | 8 | 8 |  |
|  |  | Other (specify)  …………………………………………………….. | 9 | 9 |  |
|  |  | DK | 0 | 0 |  |

| Q8 | What factors are important to you in continuing to see your regular GP?  **DO NOT PROMPT, RECORD FIRST AND OTHER MENTIONS** |  | **First**  **SC** | **Others**  **MC** |
| --- | --- | --- | --- | --- |
|  |  | Able to **make appointment** to see my GP | 1 | 1 |
|  |  | **Good communication** - ability to talk openly / freely with my GP | 2 | 2 |
|  |  | **Convenient hours** of surgery for **my work** | 3 | 3 |
|  |  | Keeps **up to** **date on medical** matters | 4 | 4 |
|  |  | **Modern** practice **facilities** | 5 | 5 |
|  |  | **Gender** of GP | 6 | 6 |
|  |  | **Location** of surgery | 7 | 7 |
|  |  | Has **knowledge** of my past **medical history** | 8 | 8 |
|  |  | Amount of **money** you need to spend to see GP | 9 | 9 |
|  |  | **Good parking** facilities | 10 | 10 |
|  |  | Presence of **practice nurse** in the practice | 11 | 11 |
|  |  | Have a **long term** **relationship** with GP | 12 | 12 |
|  |  | My doctor spends **time** with me | 13 | 13 |
|  |  | **Trust** my GP | 14 | 14 |
|  |  | Other (specify)  …………………………………………………….. | 15 | 15 |
|  |  | DK | 0 | 0 |

| Q9 | How likely or unlikely are you to change your regular GP or practice in the next 12 months?  IF LIKELY ASK: IS THAT VERY OR FAIRLY?  IF NOT LIKELY ASK: : IS THAT VERY OR FAIRLY?  **DO NOT READ OUT. Single code** | Very likely | 1 |
| --- | --- | --- | --- |
|  |  | Fairly likely | 2 |
|  |  | Neither | 3 |
|  |  | Fairly unlikely | 4 |
|  |  | Very unlikely | 5 |

| Q10 | What factors would encourage you to change your GP?  **DO NOT PROMPT, RECORD FIRST AND OTHER MENTIONS** |  | **First**  **SR** | **Others**  **MR** |  |
| --- | --- | --- | --- | --- | --- |
|  |  | Change of **address** | 1 | 1 |  |
|  |  | Lack of **appointments** | 2 | 2 |  |
|  |  | Lack of **confidence** in GP | 3 | 3 |  |
|  |  | **Convenience** | 4 | 4 |  |
|  |  | Bad **experience** | 5 | 5 |  |
|  |  | Too **expensive** | 6 | 6 |  |
|  |  | Lack of modern **facilities** | 7 | 7 |  |
|  |  | Lack of **female or male** GPs in practice | 8 | 8 |  |
|  |  | **Gender** of GP | 9 | 9 |  |
|  |  | Regular GP **left** practice (e.g. retired, moved to another practice) | 10 | 10 |  |
|  |  | Poor surgery **opening hours** | 11 | 11 |  |
|  |  | Poor **parking** facilities | 12 | 12 |  |
|  |  | Poor **relationship** with GP | 13 | 13 |  |
|  |  | Poor **services** within the practice | 14 | 14 |  |
|  |  | Lack of **trust** in GP | 15 | 15 |  |
|  |  | Having to **wait** a long time to see GP | 16 | 16 |  |
|  |  | Other (specify)  …………………………………………………….. | 17 | 17 |  |
|  |  | None – would not change | 18 | 18 |  |

| Q11 | Have you used any of the following services in the past 12 months?  **READ OUT. Multi code** | Deputising service for GP’s (out of hours service) | 1 |
| --- | --- | --- | --- |
|  |  | Accident and emergency units | 2 |
|  |  | GP co-operatives (out of hours service) | 3 |
|  |  | None of these | 4 |

ASK IF USED DEPUTISING SERVICE IN L12M. IE CODE 1 IN Q11

| Q12a | How many times in the past year did you use the deputising service for GP’s?  **RECORD NUMBER** |  |  |  |
| --- | --- | --- | --- | --- |

ASK IF USED A&E L12M. IE CODE 2 IN Q11

| Q12b | How many times in the past year did you use the accident and emergency units?  **RECORD NUMBER** |  |  |  |
| --- | --- | --- | --- | --- |

ASK IF USED GP CO-OPS L12M. IE CODE 3 IN Q11

| Q12c | How many times in the past year did you use the GP co-operatives/ out of hours services?  **RECORD NUMBER** |  |  |  |
| --- | --- | --- | --- | --- |

ASK ALL

| Q13 | Do you think it is important or not to see the same GP each visit?  IF IMPORTANT ASK: IS THAT VERY OR FAIRLY? IF NOT IMPORTANT ASK: IS THAT NOT VERY OR NOT AT ALL IMPORTANT?  **DO NOT READ OUT. Single code** | Very Important | 1 |
| --- | --- | --- | --- |
|  |  | Fairly important | 2 |
|  |  | Neither | 3 |
|  |  | Not very important | 4 |
|  |  | Not at all important | 5 |

| Q14a | Would you think it is important or not to be officially registered with one GP practice of your choice?  IF IMPORTANT ASK: IS THAT VERY OR FAIRLY? IF NOT IMPORTANT ASK: IS THAT NOT VERY OR NOT AT ALL IMPORTANT?  **DO NOT READ OUT. Single code** | Very Important | 1 |
| --- | --- | --- | --- |
|  |  | Fairly important | 2 |
|  |  | Neither | 3 |
|  |  | Not very important | 4 |
|  |  | Not at all important | 5 |

| Q14b | And do you think it is important or not to officially register with one GP practice of your choice, if you were offered various benefits, such as:  *Continuity of care, the practice would know your medical history and you would know them; and you could be invited for screening programmes run by the practice e.g. cervical screening.*  IF IMPORTANT ASK: IS THAT VERY OR FAIRLY? IF NOT IMPORTANT ASK: IS THAT NOT AT ALL OR NOT VERY IMPORTANT?  **DO NOT READ OUT. Single code** | Very Important | 1 |
| --- | --- | --- | --- |
|  |  | Fairly important | 2 |
|  |  | Neither | 3 |
|  |  | Not very important | 4 |
|  |  | Not at all important | 5 |

| Q15 | How many times do you think you would have to visit a GP before you would consider them to be your own regular GP?  **RECORD NUMBER** |  |  |
| --- | --- | --- | --- |
|  |
|  |
|  |
|  |
|  |

| Q16 | How would you describe your health, is it..?  **READ OUT.**  **Single Code** | Very good | 1 |  |
| --- | --- | --- | --- | --- |
|  |  | Good | 2 |  |
|  |  | Fair | 3 |  |
|  |  | Poor | 4 |  |
|  |  | Very poor | 5 |  |

| Q17 | Have you a long standing illness or disability?  **DO NOT READ OUT. Single Code** | Yes | 1 |  |
| --- | --- | --- | --- | --- |
|  |  | No | 2 |  |
|  |  | Prefer not to say | 3 |  |

**ASK Q18 & Q19 IF NEVER VISITED GP. IE CODE 2 IN S1. OTHERS GO TO C1**

| Q18 | Why have you never attended a GP in Ireland?  **DO NOT PROMPT. Multi code** | Don’t like doctors | 1 |  |
| --- | --- | --- | --- | --- |
|  |  | Healthy | 2 |  |
|  |  | Never had the need | 3 |  |
|  |  | Recently moved to Ireland | 4 |  |
|  |  | Too expensive | 5 |  |
|  |  | Other (specify)  …………………………………………………….. | 6 |  |
|  |  | DK | 0 |  |

| Q19 | Although you don’t have a GP, do you have a GP that you consider to be your family doctor?  **DO NOT READ OUT** **Single code** | Yes | 1 | Close & Recruit Substitute |
| --- | --- | --- | --- | --- |
|  |  | No | 2 |  |
|  |  | DK | 3 |  |

## CLASSIFICATION DETAILS

Finally, I need to ask you a few more questions

ASK ALL

| C1 | Are you..?  **READ OUT.**  **Single Code** | Single | 1 |  |
| --- | --- | --- | --- | --- |
|  |  | Married / Co-habiting with partner | 2 |  |
|  |  | Divorced / Separated | 3 |  |
|  |  | Widowed | 4 |  |

| C2 | How many children under the age of 16 live in your household?  **DO NOT READ OUT.**  **Single Code** | 1 | 1 |  |
| --- | --- | --- | --- | --- |
|  |  | 2 | 2 |  |
|  |  | 3 | 3 |  |
|  |  | 4 | 4 |  |
|  |  | 5+ | 5 |  |
|  |  | None | 6 |  |

|  |  | **Do not commute** | 1 |  |
| --- | --- | --- | --- | --- |
| C3 | How many **miles or kilometres** do you commute to work or study?  **READ OUT 1-5.**  **Single Code** | < 1 mile (<2 km) | 2 |  |
|  |  | 1 to 3 miles (2-5 km) | 3 |  |
|  |  | 4 to 6 miles (6-10 km) | 4 |  |
|  |  | > 6 miles (>10km) | 5 |  |
|  |  | DK | 0 |  |

ASK ALL

| C4 | What is the highest level of education you have achieved 1-5?  **READ OUT 1-5.**  **Single Code** | Primary school | 1 |  |
| --- | --- | --- | --- | --- |
|  |  | Junior certificate | 2 |  |
|  |  | Leaving certificate | 3 |  |
|  |  | Trade or equivalent | 4 |  |
|  |  | Third level education | 5 |  |
|  |  | Other (specify)  …………………………………………………….. | 6 |  |

SOCIAL CLASS

| C5 | INSERT SOCIAL CLASS QUESTIONS HERE | A | 1 |
| --- | --- | --- | --- |
|  |  | B | 2 |
|  |  | C1 | 3 |
|  |  | C2 | 4 |
|  |  | D | 5 |
|  |  | E | 6 |
|  |  | F1 | 7 |
|  |  | F2 | 8 |

| C6 | Which of the following ethnic groups do you belong to? **READ OUT**  **Single Code** | **White, Irish** | 1 |  |
| --- | --- | --- | --- | --- |
|  |  | **White, Irish Travelling Community** | 2 |  |
|  |  | White, British | 3 |  |
|  |  | White, European | 4 |  |
|  |  | Other white background | 5 |  |
|  |  | **Black, Irish** | 6 |  |
|  |  | Black African | 7 |  |
|  |  | Other black background | 8 |  |
|  |  | **Asian, Irish** | 9 |  |
|  |  | Chinese | 10 |  |
|  |  | Other Asian background | 11 |  |
|  |  | Mixed race | 12 |  |
|  |  | **Other, Irish** | 13 |  |
|  |  | Other (specify)  …………………………………………………….. | 14 |  |
|  |  | Refused | 15 |  |

### THANK RESPONDENT AND CLOSE
